# Research Progress on the Mechanism of Lysosome in Myocardial Ischemia-Reperfusion Injury Based on Autophagy

**DOI:** 10.31083/j.rcm2504113

**Published:** 2024-03-26

**Authors:** Yi Li, Hui Wu, Songlin Zhang, Gang Zhou, Dong Zhang, Qingzhuo Yang, Yanfang Liu, Xiaoli Huang

**Affiliations:** ^1^Institute of Cardiovascular disease, China Three Gorges University, 443003 Yichang, Hubei, China; ^2^Department of Thoracic and Cardiac Surgery, Yichang Central People's Hospital, 443003 Yichang, Hubei, China; ^3^Clinical Research Center for Ischemic Cardiovascular Disease, 443003 Yichang, Hubei, China; ^4^Department of Cardiology, Yichang Central People's Hospital, 443003 Yichang, Hubei, China; ^5^Department of Infectious Diseases, Yichang Central People's Hospital, 443003 Yichang, Hubei, China

**Keywords:** lysosome, myocardial ischemia reperfusion injury, autophagy, lysosomal membrane protein

## Abstract

In recent years, the interaction of intracellular organelles such as mitochondria and lysosomal functions has attracted increasing attention. Recent evidence suggests that mitochondrion-lysosomal contact plays a key role in regulating lysosomal biogenesis and maintaining cellular homeostasis. Myocardial ischemia and reperfusion will lead to corresponding changes in the autophagy flux in cardiomyocytes, and lysosomes are a key link in the process of autophagy, and the fusion of lysosomes and autophagosomes is an essential link in the occurrence of autophagy. Therefore, the function and homeostasis of lysosomes also undergo different changes during myocardial ischemia and reperfusion. Lysosomal-related biological factors and membrane proteins also play different roles. This article will review the mechanism of lysosomes in myocardial ischemia-reperfusion injury and the research progress of lysosomal-related proteins.

## 1. Introduction

Acute myocardial infarction (AMI) is one of the major threats to human health [1]. Myocardial ischemia reperfusion injury (MI/RI) is the leading cause of poor prognosis of AMI reperfusion treatment, about 50% of the final area of myocardial infarction is caused by reperfusion injury [2]. The study showed that for every 5% increase in myocardial infarction size, the risk of death within 1 year in AMI patients increased by 1.19 times [3]. Autophagy is a type of self-digestion function mediated by the lysosome, which is a self-protection mechanism required for normal cell growth [4]. More and more studies have shown that autophagy plays a vital role in MI/RI, but its mode of action in the MI/RI process is relatively complex. An appropriate level of autophagy is conducive to myocardial cell survival, while excessive autophagy accelerates myocardial cell death. There is presently no effective treatment plan for MI/RI caused by excessive autophagy. Therefore, actively seeking effective targets for intervention in autophagy to reduce MI/RI has important scientific and clinical significance.

The lysosome is the central organelle which maintains cell homeostasis and plays a major role in the physiological processes of material digestion, cell clearance, removal of endogenous and foreign toxic substances, and signal transduction [5]. In the process of autophagy, lysosomes and autophagosomes fuse, and the related functions of lysosomes are decisive to the outcome of autophagy [6]. In recent years, more and more attention has been paid to the related functions of lysosomes in diseases. At present, the biogenetic mechanism and role of lysosome in MI/RI autophagy remain to be further studied and clarified. This article reviews the mechanism of lysosomes in the autophagy of MI/RI cells and the research progress of lysosomal-related proteins.

## 2. Cardiomyocyte and MI/RI

### 2.1 Autophagy in MI/RI

**Fig. 1.**
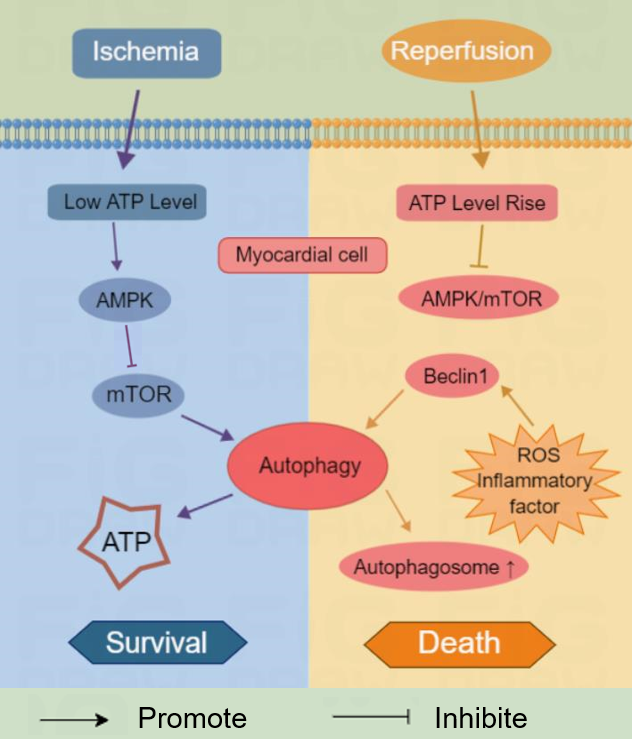
**Autophagy plays different roles of “promoting survival” and “promoting death” in different stages of myocardial ischemia and reperfusion**. mTOR, mammalian target of rapamycin; ROS, reactive oxygen species; ATP, adenosine triphosphate; AMPK, adenosine monophosphate-activated protein kinase.

Autophagy is an essential physiological function of cell self-digestion and compensation. It relies on the lysosomal mechanism to remove damaged or senescent organelles and foreign substances swallowed by cells to maintain normal cell function and metabolism [4]. Hypoxia, redox stress, nutritional stress and mitochondrial damage can induce autophagy [7]. Autophagy flux refers to the entire process of autophagy production and development, which includes three steps: (1) the initiation and nucleation of autophagosomes, (2) the expansion and formation of autophagosomes, and (3) lysosomal degradation [8]. Dysregulation of autophagy induces cell death, that is autosis [9]. The results showed that autophagy played a dual role in “promoting survival” and “promoting death” in diverse stages of MI/RI [10]. (Refer to Fig. 1) The reason for this phenomenon may be the different dependence pathways of autophagy during ischemia and reperfusion. In the stage of myocardial ischemia, insufficient exogenous energy supply can cause cardiomyocytes to supply amino acids and fatty acids to maintain cell survival through adenosine monophosphate-activated protein kinase (AMPK) mediated mammalian target of rapamycin (mTOR) dependent autophagy of rapamycin, which has A cell protection effect [11]. In the reperfusion stage, the recovery of energy supply inhibited the AMPK/mTOR signaling pathway, but inflammation and oxidative stress-induced excessive autophagy cell death in cardiomyocytes by mediating the up-regulation of Beclin-1 expression of autophagy-related protein [12].

Our previous studies have shown that cellular FLICE-like inhibitory protein (cFLIP) can inhibit Beclin-1 protein expression to inhibit autophagy cell death, and can significantly reduce myocardial cell death and myocardial tissue damage induced by hypoxia and reoxygenation [13]. Interestingly, myocardial ischemic preconditioning can up-regulate the mRNA level and protein expression of uncoupling protein 2 (UCP2) localized in mitochondria to regulate the level of mitochondrial autophagy, which can alleviate the mitochondrial dysfunction and myocardial injury caused by MI/RI. MI/RI can up-regulate the protein expression of Microtubule-Associated Protein 1 Light Chain 3-II (LC3-II) and P62 at the same time, while ischemic preconditioning + MI/RI can up-regulate LC3-II but down-regulate P62 [14]. Since the conversion of LC3-I to LC3-II represents the formation of autophagosomes, P62 binding to ubiquitinated proteins can mediate the degradation of autophagosomes and lysosomes after fusion. According to the results, both up-regulation and down-regulation of autophagy by different intervention targets can exert a protective myocardial function. The reason may be that MI/RI mediates the large accumulation of autophagy bodies, leading to autophagy dysregulation and thus autophagy cell death. However, the specific mechanism of cell death remains unclear. Dense autophagosomes can interfere with cell function by mechanically destroying organelles and interrupting cell transport, and excessive accumulation of autophagosomes is accompanied by a reduction in endoplasmic reticulum and other intimal systems and organelle dysfunction [15]. However, these observations could also be due to impaired autophagosome degradation, so more research will be needed to directly link autophagosome accumulation to cellular dysfunction.

### 2.2 Reactive Oxygen Species (ROS)/NOD-like receptor thermal protein domain associated protein 3 (NLRP3) Signaling Pathway and MI/RI

ROS is a general term for a large class of oxidants from molecular oxygen, which is the product of cell metabolism, including O2-, H2O2, OH, etc. [16]. Under normal circumstances, ROS as a natural by-product of oxygen metabolism, is at a low level in the body, as a “redox messenger” involved in intracellular signaling and regulation, and plays an important role in the cell cycle, gene expression, and maintenance of environmental homeostasis in the body [17]. When the body is subjected to harmful stimuli, ROS levels rise sharply, and the body’s oxidation-antioxidant imbalance leads to oxidative stress. Oxidative stress can damage DNA, peroxidation of lipids, change protein structure and function, and eventually destroy cell membranes and cause cell death [18].

Studies have shown that ROS is a special driver of MI/RI injury mechanisms [19]. The oxidative stress of mitochondria is mild during ischemia, but after reperfusion, ROS is produced in large quantities and rapidly promotes the oxidative stress response, causing oxidative damage and even cell death of cardiomyocytes [20]. In contrast, the use of antioxidants significantly reduced MI/RI damage [21]. The NLRP3 inflammasome is a key protein mediating inflammatory response, which can be activated by lysosomal destruction, mitochondrial stress, and other factors, and studies have shown that the NLRP3 inflammasome is also involved in MI/RI injury [22]. Importantly, ROS can stimulate the activation of the NLRP3 inflammasome, which in turn can stimulate ROS, which is undoubtedly a huge blow to MI/RI [23, 24]. Moreover, ROS may continue to rise during myocardial remodeling in the late MI/RI period [25]. Therefore, ROS plays a harmful role in MI/RI through distinct mechanisms, and targeting ROS may be a good choice to improve the prognosis of MI/RI.

### 2.3 Lysosomal Homeostasis and MI/RI-Induced Autophagy

Lysosomes are a key subcellular organelle in the process of autophagy, and the fusion of lysosomes and autophagosomes is a necessary link in the occurrence of autophagy. After the autophagosome wraps macromolecular proteins or organelles, it can fuse with the lysosome to degrade the contents of the autophagosome to produce amino acids and fatty acids to keep cell metabolism and repair after injury [6]. Lysosomes remove and recycle long-lived or damaged mitochondria via the PTEN induced putative kinase 1 (PINK1)/Parkin-mitochondrial autophagy receptor-dependent pathway. However, lysosomes may sustain damage after myocardial infarction, which may in turn cause a large number of autophagosomes in cardiomyocytes to fail to fuse with lysosomes and accumulate in the cells to promote autophagic death [26]. Mechanistic studies have shown that abnormal mitochondria-lysosome contact occurs 3 days after myocardial infarction, which can lead to lysosome enlargement and subsequently disable lysosomal clearance of damaged mitochondria [27]. In addition, sustained lysosomal injury was accompanied by a large number of apoptosis, and a large amount of P62 protein was expressed on the extracorporeal membrane. This mechanism has been shown to promote programmed necrosis key protein-crosslinked protein kinase receptor 3 ((receptor interacting protein kinase 3 (RIPK3)) phosphorylation activation, leading to mediated programmed necrosis [28]. The impaired lysosome function can also cause the clearance of damaged mitochondria, leading to the opening of the mitochondrial membrane permeability transition pore, and the release of pro-apoptotic, pro-inflammatory cytokines and ROS [29]. Several recent studies have demonstrated that promoting lysosomal biogenesis or functional homeostasis can restore autophagy flow and significantly reduce MI/RI-induced autophagy dysfunction [30, 31].

For example, Yang *et al*. [32] used Vaspin (Visceral adipose tissue-derived serine protease inhibitor) to alleviate myocardial ischemia/reperfusion injury by activating autophagy flux and restoring lysosome function, restoring damaged lysosome function during reperfusion, eliminating excessive accumulation of damaged proteins and organelles wrapped in autophagy during reperfusion, to restore autophagy flow and improve cardiac function after reperfusion. Therefore, lysosomal homeostasis may be the key to the dual “pro-survival” and “pro-death” role of autophagy in MI/RI. In the process of MI/RI, mammalian target of rapamycin complex 1 (mTORC1), as the target of signaling in the central mechanism in lysosomes, is an important negative regulator of autophagy, and its activation depends on the activation of mTOR at the position of mTORC1 on lysosomes, which in turn negatively regulates autophagy and lysosomes at the translational and post-translational levels, thus regulating autophagy formation and lysosomal degradation [33]. Lysosomes have become increasingly prominent as nutrient signaling hubs, linking autophagy with the mTORC1/transcription factor EB (TFEB) pathway. However, the regulatory role of lysosomal membrane proteins in this pathway has not been fully elucidated and needs to be further studied and explored.

## 3. Lysosomal-Associated Proteins and MI/RI

### 3.1 TFEB and MI/RI

TFEB is a member of the basic helix-loop-helix leucine zipper transcription factor family [34]. It consists of 476 amino acid residues and is an essential protein regulating lysosome homeostasis and autophagy [35]. TFEB is localized in the cytoplasm in its inactivated state. When cells are stimulated by lysosomal stress and hunger, TFEB phosphorylation enters the nucleus and binds to lysosomal gene network CLEAR (coordinated lysosomal expression and regulation), which promotes the expression of lysosomal gene network and ultimately enhances lysosomal biogenesis and autophagy [35, 36]. In addition, TFEB also promotes the expression of autophagy-related genes including Sequestosome (SQSTM1), UV radiation resistance associated (UVRAG), WD-repeat protein interacting with phosphoinosides (WIPI), autophagy-related protein 9B (ATG9B), LC3, etc. [35, 37]. In addition to TFEB participating in lysosomal biogenesis and autophagy formation, TFEB can also promote the fusion of autophagosomes and lysosomes [35]. In addition, TFEB-mediated autophagy can decrease mitochondrial dysfunction, on the contrary, the imbalance of TFEB may lead to mitochondrial dysfunction and autophagy flow block [38]. In short, TFEB regulates autophagy by targeting all aspects of the autophagy pathway.

In the process of MI/RI, TFEB is activated and translocated to the nucleus, which in turn up-regulates the genes affected by autophagy and lysosome function. The autophagy flux of cardiomyocytes increased by TFEB overexpression and decreased after TFEB was down-regulated by specific small interfering RNA (siRNA) [39]. Li *et al*. [40] explored the role and mechanism of cilostazol in rat MI/RI model and found that cilostazol promoted nuclear ectopia of TFEB and promoted the expression of lysosomal-associated membrane protein-1 (LAMP1), LAMP2, cathepins B and cathepins D, increased the ratio of LC3II/LC3I and decreased p62 abundance. The TFEB inhibitor temsirolimus(CCI-779) reversed the effect of cilostazol. These data suggest that the protective effect of cilostazol on MI/RI is achieved by increasing the transcriptional activity of TFEB. Many studies have proved that it is a feasible method to promote autophagy flow of cardiomyocytes by increasing the expression of TFEB. It is worth noting that recent studies by Pan *et al*. [33] have shown that when the autophagy flux is inhibited in the later stage of MI/RI, the activation of TFEB will induce the accumulation of autophagosomes in cardiomyocytes and promote cellular autophagic death of myocardial cells, which will aggravate myocardial infarction injury, while inhibition of endogenous TFEB can alleviate the process of MI/RI. This study indicates that TFEB may be a double-edged sword in MI/RI. Fortunately, He *et al*. [41] proved that exendin-4 restored the blocked autophagy flux by promoting the nuclear translocation of TFEB. The recovery of autophagy flux helps to cut down oxidative stress, maintain mitochondrial function, reduce infarct size, and maintain cardiac function. In addition, Liu *et al*. [42] found that the increase of TFEB enhanced the expression of autophagy-related genes in endothelial cells and promoted angiogenesis and cardiac function recovery after myocardial infarction (MI). These findings suggest that TFEB plays a significant role in autophagy.

### 3.2 Lysosomal Transient Receptor Potential Mucolipin 1 (TRPML1) and MI/RI

TRPML1/MCOLN1 (mucolipin TRP cation channel 1) is a non-selective cation channel located in lysosomes and belongs to the TRPML1-3 (The mucolipin subfamily of TRP channels) subfamily of TRP channels. TRPML1 is widely distributed in various tissues and cells, while TRPML2 and TRPML3 are only expressed in special cell types (Mucolipins: Intracellular TRPML1-3 channels). TRPML1 is involved in a variety of cellular reactions, such as endocytosis, exocytosis, and autophagy. It is a fundamental regulator of most lysosome transport processes and is necessary to maintain lysosome function and cell homeostasis [43, 44].

TRPML1 induces the release of lysosomal zinc into the cytoplasm, which destroys the fusion between autophagosomes and lysosomes, inhibits the turnover of mitochondria, and accumulates a large number of damaged mitochondria, which leads to more ROS release, which in turn stimulates TRPML1 channels, finally leading to the inhibition of autophagy flux [45]. At present, several studies have proved that TRPML1 is an important part of the occurrence and development of some cardiovascular diseases. For example, in the study of Pan *et al*. [46], Systemic proteasome inhibition (PSMI) activated TFEB in cardiomyocytes and its downstream signals depended on the activation of TRPML1. In the study of Xing *et al*. [47], TRPML1 is directly involved in the blocking of myocardial autophagy flux after MI/RI. It is generally known that a large amount of ROS is produced during the reperfusion of MI/RI. Stimulated by ROS, TRPML1 mediates the release of lysosomal zinc and prevents the fusion between autophagosomes and lysosomes, followed by a large amount of mitochondrial accumulation, which finally blocks the autophagy flux of cardiomyocytes. When TRPML1 was down-regulated, these phenomena were reversed *in vivo* and *in vitro*, the blocked myocardial autophagy flux was restored, and the infarct area of mouse MI/RI was markedly reduced [47]. Now, there is not much research on TRPML1 in MI/RI, but combined with these existing research results, it is a very promising strategy to treat TRPML1 as a treatment of MI/RI.

### 3.3 lysosomal-associated transmembrane protein 4B (LAPTM4B) and MI/RI

LAPTM4B is located on the lysosomal membrane and is a four-transmembrane protein, mainly localized to late endosomes and lysosomes [48]. The LAPTM4B gene, located on chromosome 8q22.1, was first found in human hepatocellular carcinoma. In humans, there are two protein isoforms, LAPTM4B-35 and LAPTM4B-24, but only LAPTM4B-24 was found to be highly expressed in the myocardium in mice [49]. LAPTM4B is thought to be involved in the regulation of autophagy through the epidermal growth factor receptor (EGFR) pathway. EGFR regulates autophagy by activating PI3K/AKT/mTOR, EGFR-rat sarcoma (EGFR-RAS), EGFR-Beclin 1, and EGFR-signal transducer and activator of transcription 3 (EGFR-STAT3) signaling pathways. PI3K, AKT, and mTOR are downstream molecules of the EGFR signaling pathway, which negatively regulate autophagy by inhibiting the autophagy-activated kinase 1 complex [49]. Gu *et al*. [50] recently established a mouse MI/RI model, and its expression was down-regulated in the heart of mice injured by I/R (30 minutes/2 hours) and in neonatal rat cardiomyocytes injured by hypoxia/reoxygenation (6 hours/2 hours). The results confirmed that LAPTM4B down-regulation aggravated MI/RI through unmatched activation of mTORC1 signaling and inhibition of TFEB activity, while LAPTM4B up-regulation had a cardioprotective effect by restoring autophagy flux to protect cardiomyocytes from I/R injury. Whether LAPTM4B can be upregulated by a drug to protect the heart from I/R damage remains to be further investigated.

### 3.4 LAMPs and MI/RI

LAMPs can be divided into LAMP1 and LAMP2, which are the main protein components of lysosomal membrane. LAMP1 is a type I transmembrane protein, mainly distributed in the lysosomal membrane and endocytosome membrane, and is usually used as a lysosomal marker to maintain lysosomal function [51]. LAMP1 is a highly glycosylated lysosomal membrane protein that is thought to protect lysosomal membranes from intracellular proteolysis [52]. Recent studies have discovered that LAMP1 plays a new role in MI/RI. According to the results of Song *et al*. [53] in myocardial I/R injury, LAMP1 is down regulated. LAMP1 is a receptor for tumor necrosis factor related protein 3 (CTRP3), and it can interact with CTRP3. The protein expression level of LAMP1 is positively correlated with CTRP3. Upregulation of CTRP3 can protect myocardium, reduce myocardial apoptosis, alleviate myocardial injury, and reduce myocardial infarction area by activating the LAMP1/c-jun amino terminal kinase interacting protein 2 (JIP2)/c-jun N-terminal kinase (JNK) pathway. The mechanism is that the upregulation of CTRP3 leads to overexpression of LAMP1. The reduction of MI/RI injury was achieved, while the levels of cardiac troponin I (cTnI), serum creatinine phosphokinase (CPK), lactate dehydrogenase (LDH), and caspase-3 were also correspondingly reduced.

## 4. Conclusions

More and more studies have shown that lysosomes play a central role in MI/RI. Damage of lysosome homeostasis leads to decreased autophagic flow activity, autophagosome accumulation, and autophagic cell death. TFEB is the master gene of lysosomal biogenesis, coordinating this process by driving the expression of autophagy and lysosomal genes and linking autophagy to lysosomal biogenesis. TRPML 1 can regulate various cellular activities, including autophagy, lysosomal exocytosis, apoptosis, lipid transport, and exosome release, which is the key to regulating lysosomal biogenesis. In the future, specific drugs targeting TRPML1 may be put in place to prevent and treat MI/RI. The LAPTM4B-mediated signaling pathway can ultimately regulate the autophagy flux of cardiomyocytes, thereby affecting the survival and death of cardiomyocytes after MI/RI. LAMP1, as a constituent protein and important marker of lysosomes, promotes overexpression of LAMP and may become a new strategy for restoring lysosomal function in M/IRI. In short, lysosomal function is closely related to autophagy in MI/RI, and future research based on lysosomal homeostasis can provide new diagnostic and therapeutic ideas for the prevention and treatment of MI/RI in clinical practice.
